# Subclinical median nerve alterations after distal radius fractures: a comparative analysis of volar plate fixation and cast immobilization using dynamic ultrasonography and electrophysiology

**DOI:** 10.1007/s00402-026-06364-7

**Published:** 2026-05-27

**Authors:** Sefa Erdem Karapinar, Esma Arslan, Furkan Cagri Oguzlar, Ahmet Yunus Hatip, Vedat Onur Akgun, Recep Dincer, Sanem Asci, Volkan Kizilkaya

**Affiliations:** 1 Department of Orthopedics and Traumatology, Süleyman Demirel University, Isparta, Turkey; 2 Department of Sports Medicine, Süleyman Demirel University, Isparta, Turkey; 3 Department of Emergency Medicine, Süleyman Demirel University, Isparta, Turkey; 4 Department of Neurology, Süleyman Demirel University, Isparta, Turkey; 5 Department of Orthopedics and Traumatology, Private Metrolife Hospital, Sanliurfa, Turkey

**Keywords:** Distal radius fracture, Median nerve, Volar plate fixation, Ultrasonography

## Abstract

**Background:**

Distal radius fractures may affect the median nerve through trauma-related and treatment-related mechanisms. While overt neurological deficits are uncommon, subclinical median nerve alterations may occur following both conservative and surgical treatment.

**Purpose:**

To determine whether treatment modality influences subclinical median nerve behavior using combined dynamic ultrasonographic and electrophysiological assessment.

**Methods:**

This retrospective observational study included 42 patients with unilateral distal radius fractures. 18 patients were treated conservatively with circular casting, and 24 patients underwent volar plate fixation. Ultrasonographic assessment of the median nerve was performed at the distal radius level in neutral wrist position, as well as during wrist flexion and extension, using a dynamic ultrasonographic approach, and was combined with electrophysiological evaluation including sensory and motor nerve conduction studies. In the surgically treated group, only patients with Soong type 1 or type 2 plate positioning were included.

**Results:**

Clinical functional outcome scores were comparable between groups, despite a higher frequency of mild clinical signs such as positive Tinel test and night pain in the plate group. However, ultrasonographic evaluation demonstrated significantly greater position-dependent median nerve enlargement and flattening in the volar plate group, particularly during wrist flexion and extension (*p* < 0.05). Electrophysiological assessment revealed a significant reduction in sensory nerve conduction velocity on the affected side in surgically treated patients compared with the cast group (*p* < 0.05), while motor conduction parameters remained preserved. These differences were observed despite comparable clinical functional outcomes between groups.

**Conclusion:**

Patients treated with volar plate fixation demonstrated more pronounced subclinical ultrasonographic and electrophysiological median nerve alterations compared with those treated conservatively with cast immobilization. However, given the retrospective and non-randomized design, these findings should be interpreted as associative rather than causal. Combined ultrasonographic and electrophysiological assessment may be useful for detecting early median nerve involvement and guiding follow-up evaluation, even in the absence of clinically overt symptoms.

## Introduction

 Distal radius fractures are among the most commonly encountered fracture types in orthopedic practice. Their incidence increases markedly in the elderly population, particularly in association with osteoporosis [1, 2]. In the treatment of these fractures, either conservative cast immobilization or surgical treatment methods are preferred based on fracture type, stability, and patient-related factors. In recent years, volar locking plate–screw systems have gained widespread use, especially in unstable distal radius fractures, as they allow early mobilization and facilitate anatomical reduction [3]. However, distal radius fractures and the treatment methods applied may lead to increased carpal tunnel pressure at the wrist level, resulting in median nerve involvement. Trauma-related edema, hematoma, displacement of fracture fragments, immobilization with cast application, and implants placed in the volar region during surgery may cause mechanical and physiological effects on the median nerve [4, 5]. The incidence of median nerve dysfunction and carpal tunnel syndrome following distal radius fractures has been reported in the literature to range between 0.5% and 14.6% [6, 7]. In patients treated with volar plate fixation, the distal placement of the plate is of critical importance with respect to soft tissues and neural structures. Placement of the plate beyond the volar rim of the distal radius increases the risk of pressure on the flexor tendons and the median nerve. The Soong classification is widely used to assess volar plate prominence. Soong type 1 and particularly type 2 cases represent situations in which the plate crosses the critical line and the risk to soft tissues is increased [8]. Although classical clinical examination findings and patient-reported symptoms are guiding tools in the evaluation of the median nerve, they may be insufficient for detecting early structural changes. Ultrasonography is a non-invasive and dynamic method that has been increasingly used in the evaluation of peripheral nerves and allows objective measurement of the median nerve cross-sectional area (MNCSA) and shape alterations [9]. It has been reported that structural enlargement of the median nerve can be detected before the emergence of clinical and electrophysiological findings and may serve as an indicator of early compression [10]. Electromyography (EMG) is one of the gold standard methods for evaluating the functional status of the median nerve and provides objective data through nerve conduction velocity, distal motor latency, and action potential amplitudes [11]. The combined assessment of ultrasonographic morphological changes and EMG enables a more reliable identification of subclinical median nerve involvement [12]. Although studies investigating the effects of volar plate fixation on the median nerve are available in the literature, studies directly comparing patients treated conservatively with circular cast immobilization and those treated surgically are quite limited. However, in patients treated with cast immobilization, factors such as edema, wrist position, and duration of immobilization may also increase median nerve pressure [13]. This situation necessitates clarification of whether median nerve involvement is exclusively related to surgical treatment or represents a general consequence of distal radius fractures.

In the literature, evaluations have generally been limited to either ultrasonographic or electrophysiological assessment alone, and comprehensive analyses incorporating clinical functional outcomes have often remained limited. There is an ongoing need for comparative studies in which the structural, functional, and clinical outcomes of the median nerve are evaluated together. To our knowledge, no previous study has simultaneously evaluated dynamic position-dependent ultrasonographic changes together with electrophysiological findings in a comparative setting of surgical and conservative treatment for distal radius fractures. Most previous studies have focused on either static imaging or isolated electrophysiological parameters, without integrating these modalities or considering the potential role of dynamic nerve behavior. This represents a significant gap in the current literature. Accordingly, the present study aimed to comparatively evaluate structural and functional median nerve changes in patients with distal radius fractures treated with circular cast immobilization or volar plate fixation using combined ultrasonographic, electrophysiological, and clinical assessments.

## Materials and methods

### Study design and patient selection

This single-center, retrospective, observational study included patients diagnosed with distal radius fractures who presented to the emergency department or the orthopedics and traumatology outpatient clinic and received either conservative or surgical treatment. Medical records of patients treated between January 2024 and January 2025 were retrospectively analyzed. A total of 42 patients were included. Data were obtained from the hospital information management system, clinical examination records, radiological images, ultrasonography reports, and electrophysiological examination results.

Inclusion criteria were as follows: Age ≥ 18 years at the time of injury; Diagnosis of an acute, unilateral distal radius fracture confirmed by radiographic evaluation; Treatment with either circular cast immobilization or volar plate fixation according to routine clinical and radiological criteria; Availability of complete clinical, ultrasonographic, and electrophysiological follow-up data at 12 months post-injury.

For the surgically treated group, only patients with Soong type 1 or type 2 volar plate positioning were included to ensure homogeneity of implant-related factors.

Exclusion criteria included pathological or malignancy-related fractures; bilateral distal radius fractures or concomitant fractures in the same extremity; a prior history of carpal tunnel syndrome, median nerve neuropathy, or peripheral nerve disease of the upper extremity; a history of tumors, inflammatory arthritis, or gout affecting the hand or wrist; development of marked malunion or postoperative deformity during fracture healing; severely comminuted fractures in which anatomical landmarks could not be reliably identified during ultrasonographic or electrophysiological assessment. This exclusion was applied to ensure reliable and reproducible ultrasonographic and electrophysiological measurements; incomplete clinical follow-up or irregular attendance at scheduled controls. Patients with Soong type 0 plate positioning were excluded because minimal plate prominence may reduce the ability to detect potential implant-related effects on the median nerve and increase heterogeneity within the surgical cohort. Treatment decisions were made according to routine clinical and radiological criteria, including fracture displacement, stability after closed reduction, risk of secondary displacement, patient age, bone quality, and functional demands. This approach reflects real-world clinical decision-making, in which treatment selection is often influenced by multiple fracture- and patient-related factors rather than classification systems alone. Fracture classification according to AO/OTA was not systematically available in the retrospective records and was therefore not included in the analysis. As expected in routine clinical practice, surgically treated fractures were likely to represent relatively more unstable fracture patterns compared with conservatively treated fractures. Therefore, the observed differences may partially reflect fracture-related factors in addition to treatment-related effects. This limitation restricts the ability to control for fracture severity as a potential confounding factor when interpreting differences between the treatment groups.

The study protocol was reviewed and approved by the Health Sciences Ethics Committee (Meeting date: 18.08.2025; Meeting No: 102; Decision No: 1) and was conducted in accordance with the current principles of the Declaration of Helsinki.

### Treatment modalities and grouping

Patients were divided into two groups according to the treatment modality:

*Cast group*: Patients treated with circular cast immobilization following closed reduction. The duration of immobilization was determined based on fracture stability and the clinical course of healing.

*Plate group*: Patients treated surgically with volar plate internal fixation for distal radius fracture using a standard volar approach. No patient underwent prophylactic carpal tunnel release.

The choice of treatment was based on fracture characteristics, stability, patient age, functional demands, and the surgeon’s clinical judgment. As treatment allocation was not randomized, the observed differences between groups may partially reflect underlying fracture severity rather than the treatment modality itself.

To reduce implant-related heterogeneity, only patients with Soong type 1 or type 2 plate positioning were included in the surgically treated cohort. The Soong classification was applied postoperatively for descriptive purposes and was not used to determine treatment allocation.

### Clinical evaluation

All patients were evaluated during follow-up for median nerve–related clinical findings. Tinel, Phalen, and Durkan tests were performed using standard techniques. The presence of night pain, paresthesia, numbness, and symptoms compatible with carpal tunnel syndrome was recorded. Patients clinically diagnosed with carpal tunnel syndrome were specifically noted.

Functional outcomes were assessed using the Mayo Wrist Score (MWS), Patient-Rated Wrist Evaluation (PRWE), and Michigan Hand Questionnaire (MHQ), which evaluate wrist function, pain severity, and limitations in daily activities.

### Ultrasonographic evaluation

Ultrasonographic examinations were performed to assess the morphology of the median nerve at the distal radius level on both the affected and unaffected sides. Measurements were obtained with the wrist positioned in neutral position, flexion, and extension. Conventional ultrasonography was performed using B-mode imaging with a GE Logic E9 ultrasound system (GE Healthcare, Chicago, IL, USA) equipped with a 15-MHz high-frequency linear-array transducer under musculoskeletal imaging settings.

The median nerve cross-sectional area (CSA), radio–ulnar diameter (D1), and dorsal–palmar diameter (D2) were measured. The difference in CSA between the affected and unaffected sides (ΔCSA) was calculated. Median nerve flattening ratios were determined as the anteroposterior-to-mediolateral (AP/ML) ratio to evaluate morphological changes across different wrist positions.

**Fig. 1:**
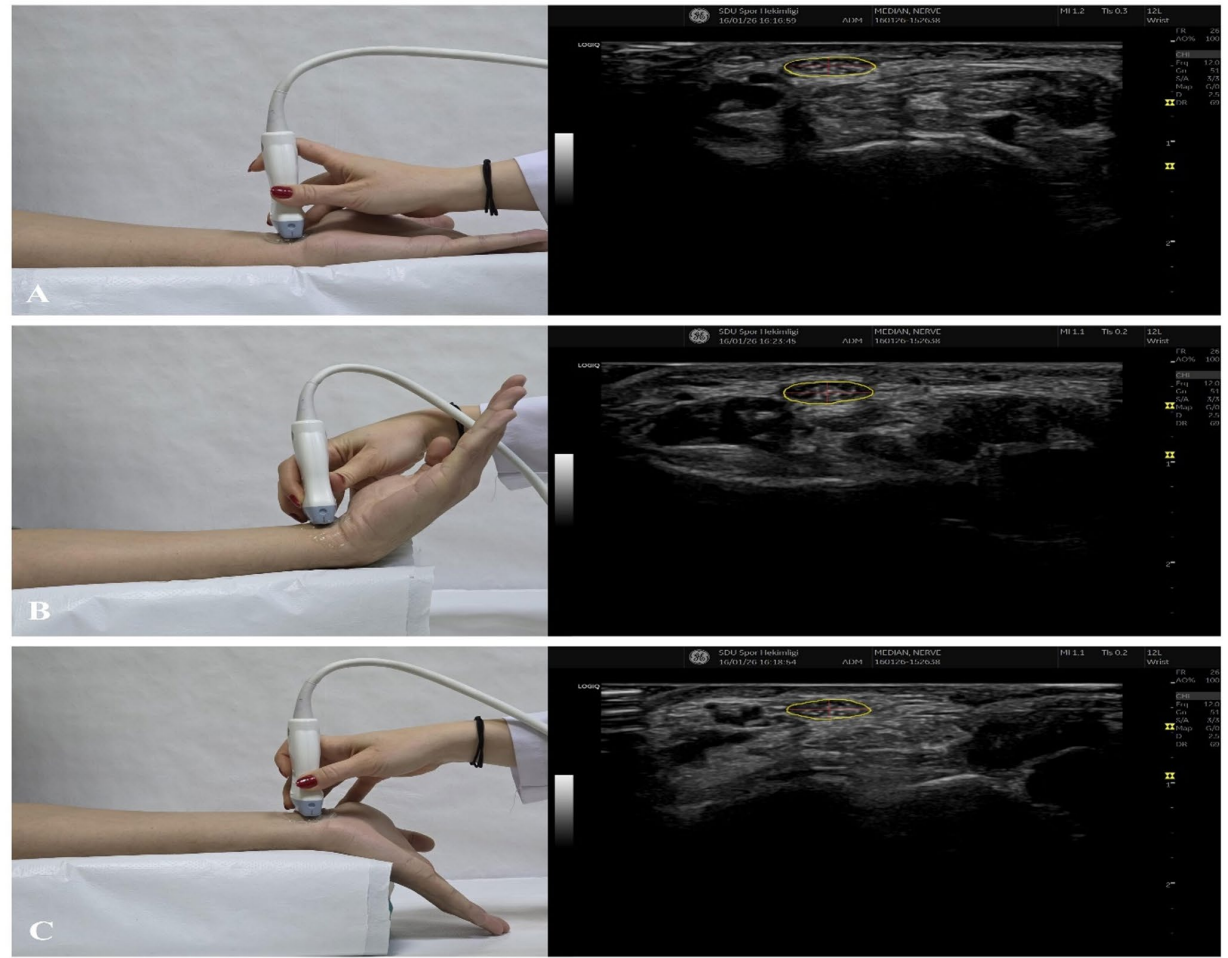
Ultrasonographic assessment of the median nerve at the distal radius level in different wrist positions.** A** Neutral wrist position, **B** wrist flexion, and **C** wrist extension. The left panels illustrate probe positioning during ultrasonographic examination, while the right panels show corresponding transverse ultrasound images of the median nerve. The median nerve is outlined for measurement. Quantitative assessment included measurement of the median nerve cross-sectional area (MNCSA), radial–ulnar diameter (**D1**), and dorsal–palmar diameter (**D2**). The white arrow indicates the position of the volar plate in surgically treated patients. All measurements were obtained at standardized anatomical landmarks and at the same level for each wrist position

All measurements were obtained using standardized anatomical landmarks, and care was taken to perform measurements at the same anatomical level for each patient. All ultrasonographic examinations were performed by an orthopedic surgeon experienced in musculoskeletal ultrasonography with approximately 5 years of clinical experience in peripheral nerve imaging. Examinations were conducted under standardized outpatient examination conditions using consistent wrist positioning protocols in order to minimize measurement variability. Representative ultrasonographic images and measurement techniques are illustrated in Fig. 1.

### Electrophysiological Evaluation

Median nerve electrophysiological properties were assessed using standard nerve conduction studies. Sensory nerve action potential amplitude, distal latency, conduction distance, and conduction velocity were recorded for both the affected and unaffected sides. Motor nerve conduction studies included measurements of compound muscle action potential amplitude, distal latency, conduction distance, and motor conduction velocity.

Electrophysiological findings were evaluated in conjunction with clinical and ultrasonographic data to compare median nerve function between treatment groups. Electrophysiological examinations were performed under standard laboratory conditions using conventional nerve conduction study protocols. Care was taken to maintain consistent environmental conditions during testing to minimize variability in nerve conduction measurements.

### Timing of follow-up assessments

All clinical, ultrasonographic, and electrophysiological evaluations were performed at the 12-month follow-up. This time point was selected to minimize the influence of acute post-traumatic edema, transient neurapraxia, and early inflammatory changes, and to allow stabilization of functional and neurophysiological outcomes, as supported by prior studies evaluating median nerve recovery following distal radius fractures [13].

### Sample size considerations

Given the retrospective design, sample size adequacy was assessed based on effect size estimates derived from the study data [14]. For the ultrasonographic parameter demonstrating the largest between-group difference - median nerve flattening ratio (AP/ML) during wrist extension (AP/ML) on the affected side- the effect size (Cohen’s d) was calculated as 1.13, indicating a large effect. Assuming a two-sided α level of 0.05, a statistical power of 80%, and a group allocation ratio consistent with the study population (plate/cast ≈ 1.33), a minimum sample size of approximately 28 patients was required. The final sample size of 42 patients was therefore considered sufficient to detect large effect sizes, while acknowledging that smaller or moderate effects, particularly in electrophysiological parameters, may require larger cohorts.

### Statistical analysis

Statistical analyses were performed using R software (R Foundation for Statistical Computing, Vienna, Austria). Continuous variables were expressed as mean ± standard deviation, and categorical variables as counts and percentages. Normality of continuous variables was assessed using visual inspection and analytical methods. Between-group comparisons were performed using the independent samples t-test for normally distributed variables; Welch’s correction was applied when variance homogeneity was not met. Categorical variables were analyzed using the chi-square test or Fisher’s exact test, as appropriate. Correlation analyses were conducted to evaluate associations between ultrasonographic median nerve parameters obtained in different wrist positions and electrophysiological measurements of the median nerve. Graphical representations were generated using GraphPad Prism software (GraphPad Software, San Diego, CA, USA).

Given the exploratory nature of the study and the limited sample size, formal correction for multiple comparisons was not applied. Therefore, the statistical findings should be interpreted cautiously. A p value < 0.05 was considered statistically significant.

## Results

A total of 42 patients with distal radius fractures were included in the study, including 24 patients treated with volar plate fixation and 18 treated with cast immobilization. Demographic characteristics, dominant side distribution, injured side, and immobilization duration were comparable between groups. Regarding median nerve–related clinical findings, positive Tinel test results and night pain were significantly more frequent in the plate group compared with the cast group (*p* = 0.002 and *p* = 0.029, respectively). Positive Phalen and Durkan tests, as well as clinically diagnosed carpal tunnel syndrome, were infrequent and did not differ significantly between treatment groups (Table 1).

**Table 1:** Baseline demographic characteristics and clinical findings according to treatment modality

Variable		Overall (*n* = 42)	Cast (*n* = 18)	Plate (*n* = 24)	*p* value
Age (years)		46,40 ± 16,80	43,80 ± 18,10	48,40 ± 15,80	0,383
Sex (n, %)	**Male**	16 (38,1)	8 (44,4)	8 (33,3)	0,680
	**Female**	26 (61,9)	10 (55,6)	16 (66,7)	
Immobilization duration (days)		32,20 ± 6,10	30,70 ± 6,00	33,30 ± 6,00	0,161
Dominant side (right)		40 (95,2)	16 (88,9)	24 (100,0)	0,178
Injured side (left)		26 (61,9)	12 (66,7)	14 (58,3)	0,819
Tinel test		10 (23,8)	0 (0,0)	10 (41,7)	**0**,002*
Phalen test		2 (4,8)	0 (0,0)	2 (8,3)	0,498
Durkan test		4 (9,5)	0 (0,0)	4 (16,7)	0,122
Night pain		6 (14,3)	0 (0,0)	6 (25,0)	**0**,029*
Carpal tunnel syndrome diagnosis		2 (4,8)	0 (0,0)	2 (8,3)	0,498

Values are presented as mean ± standard deviation or number (%), as appropriate. Comparisons between the cast and plate groups were performed using the independent samples t-test for continuous variables and the chi-square or Fisher’s exact test for categorical variables. *:significant value

**Fig. 2:**
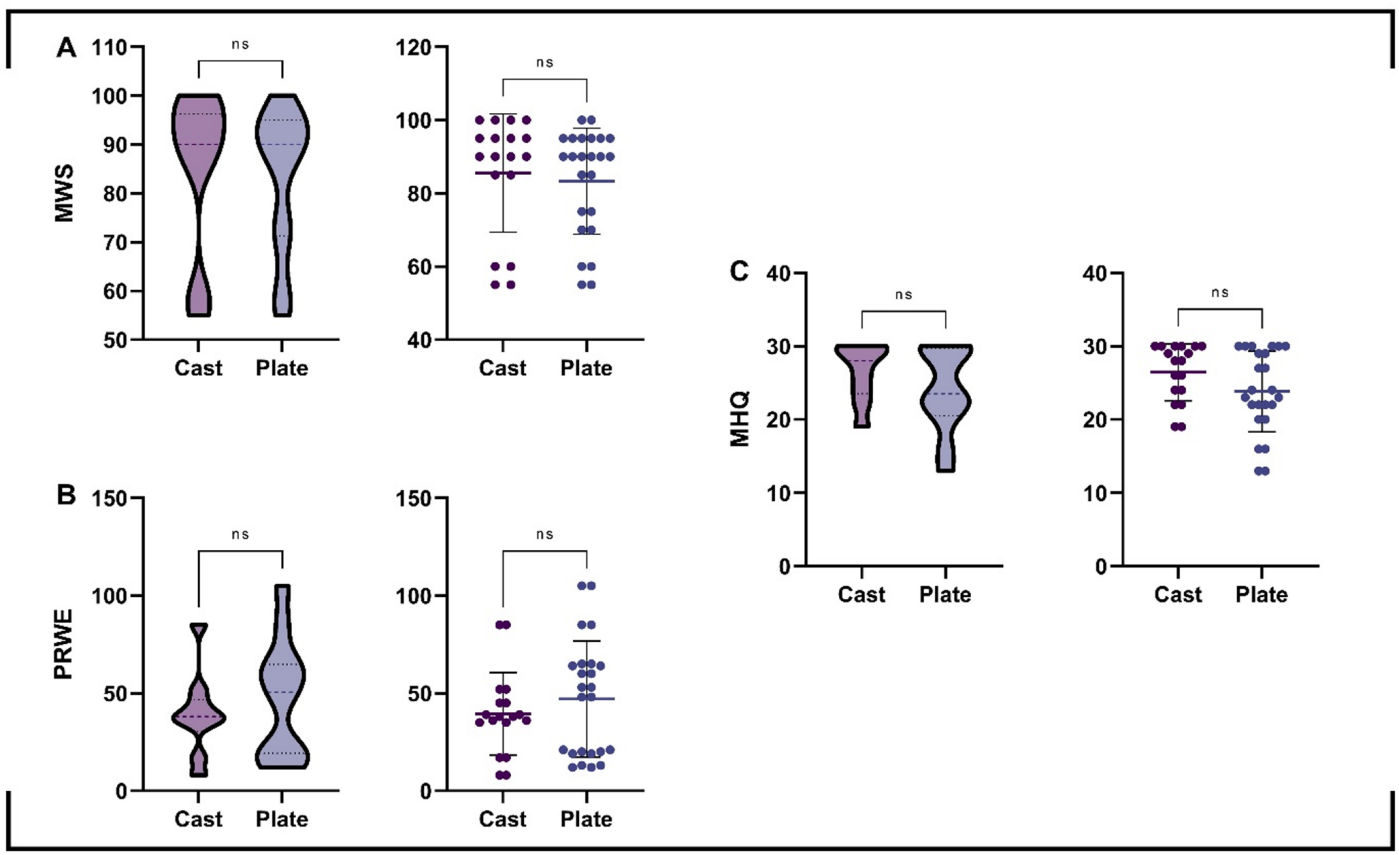
Distribution of clinical outcome scores according to treatment modality. Violin plots illustrating the distribution of ***A*** *Mayo Wrist Score (MWS)*, ***B*** Patient-Rated Wrist Evaluation (PRWE),* and* ***C*** Michigan Hand Questionnaire (MHQ) scores in patients treated with cast or plate fixation. Individual data points are overlaid on each violin plot, with central horizontal lines indicating the median values and error bars representing the interquartile range.

Clinical outcome scores, including the Mayo Wrist Score (MWS), Patient-Rated Wrist Evaluation (PRWE), and Michigan Hand Questionnaire (MHQ), were comparable between the cast and plate groups, with no statistically significant differences observed (all *p* > 0.05). The distribution of clinical scores is illustrated in Fig. 2.

Ultrasonographic evaluation demonstrated significantly greater position-dependent median nerve enlargement and flattening in the plate group, particularly during wrist flexion and extension. The most prominent differences were observed in affected-side median nerve cross-sectional area (CSA), flattening ratio, and dorsal–palmar diameter (D2) measurements. In contrast, radial–ulnar diameter (D1) and ΔCSA measurements were generally comparable between groups. In the neutral wrist position, affected-side dorsal–palmar diameter (D2) remained significantly greater in the plate group (Table 2).

**Table 2:** Ultrasonographic assessment of the median nerve in neutral, flexion, and extension wrist positions according to treatment modality

Variable		Overall (*n* = 42)	Cast (*n* = 18)	Plate (*n* = 24)	*p* value
NP ΔCSA (affected − unaffected)		0.01 ± 0.01	0.00 ± 0.01	0.01 ± 0.02	0.103
NP flattening ratio (AP/ML)		0.34 ± 0.10	0.32 ± 0.06	0.36 ± 0.12	0.233
NP D1 (radio–ulnar)	Affected	0.68 ± 0.11	0.65 ± 0.09	0.71 ± 0.12	0.060
	Unaffected	0.63 ± 0.09	0.62 ± 0.09	0.64 ± 0.08	0.532
NP D2 (dorsal–palmar)	Affected	0.23 ± 0.05	0.20 ± 0.02	0.25 ± 0.06	0.001*
	Unaffected	0.23 ± 0.08	0.25 ± 0.10	0.23 ± 0.05	0.472
Median nerve CSA – wrist flexion (cm²)	Affected	0.11 ± 0.03	0.10 ± 0.02	0.12 ± 0.03	0.024*
	Unaffected	0.10 ± 0.02	0.09 ± 0.02	0.11 ± 0.02	0.070
Median nerve ΔCSA – wrist flexion ΔCSA (affected − unaffected)		0.01 ± 0.01	0.00 ± 0.01	0.01 ± 0.02	0.103
Median nerve flattening ratio – wrist flexion (AP/ML)		0.41 ± 0.11	0.38 ± 0.09	0.44 ± 0.13	0.110
Median nerve D1 – wrist flexion (radio–ulnar)	Affected	0.60 ± 0.08	0.59 ± 0.08	0.61 ± 0.08	0.422
	Unaffected	0.56 ± 0.09	0.53 ± 0.10	0.58 ± 0.07	0.053
Median nerve D2 – wrist flexion (dorsal–palmar)	Affected	0.25 ± 0.06	0.22 ± 0.03	0.27 ± 0.07	0.025*
	Unaffected	0.25 ± 0.06	0.26 ± 0.04	0.24 ± 0.07	0.307
Median nerve CSA – wrist extension (cm²)	Affected	0.11 ± 0.03	0.10 ± 0.01	0.12 ± 0.03	0.014
	Unaffected	0.10 ± 0.03	0.09 ± 0.02	0.11 ± 0.03	0.017
Median nerve ΔCSA – wrist extension (affected − unaffected)		0.01 ± 0.01	0.01 ± 0.01	0.01 ± 0.01	0.868
Median nerve flattening ratio – wrist extension (AP/ML)		0.34 ± 0.12	0.27 ± 0.06	0.39 ± 0.13	0.001*
Median nerve D1 – wrist extension (radio–ulnar)	Affected	0.65 ± 0.09	0.66 ± 0.09	0.64 ± 0.09	0.535
	Unaffected	0.63 ± 0.09	0.61 ± 0.11	0.65 ± 0.06	0.209
Median nerve D2 – wrist extension (dorsal–palmar)	Affected	0.22 ± 0.05	0.19 ± 0.02	0.25 ± 0.06	< 0.001*
	Unaffected	0.21 ± 0.07	0.19 ± 0.05	0.23 ± 0.07	0.083

Ultrasonographic parameters are presented as mean ± standard deviation. Comparisons between cast and plate groups were performed using the independent samples t-test (Welch correction applied when variance homogeneity was violated).

*NP* neutral position, *CSA* cross-sectional area, *ΔCSA* difference between affected and unaffected sides, *AP* anteroposterior diameter, *ML* mediolateral diameter, *D1*, radio–ulnar diameter, D2, dorsal–palmar diameter. Ultrasonographic measurements were obtained from the median nerve in the neutral wrist position as well as during wrist flexion and extension.

*significant value

Electrophysiological assessment of the median nerve revealed limited differences between treatment groups. Sensory nerve conduction velocity on the affected side was significantly lower in the plate group compared with the cast group (43.82 ± 4.97 vs. 47.78 ± 6.33 m/s, *p* = 0.028). In addition, sensory nerve conduction distance on the unaffected side was greater in the plate group than in the cast group (138.25 ± 11.86 vs. 129.72 ± 8.88 mm, *p* = 0.014).

No significant between-group differences were observed in the remaining sensory or motor electrophysiological parameters (Table 3).

**Table 3:** Electrophysiological assessment of the median nerve according to treatment modality

Variable		Overall (*n* = 42)	Cast (*n* = 18)	Plate (*n* = 24)	*p* value
Sensory amplitude (µV)	Affected	28.00 ± 8.00	27.73 ± 7.30	26.46 ± 7.05	0.572
	Unaffected	31.29 ± 13.42	31.40 ± 11.75	31.20 ± 14.63	0.962
Sensory latency (ms)	Affected	2.81 ± 0.26	2.82 ± 0.29	2.80 ± 0.24	0.825
	Unaffected	2.65 ± 0.45	2.65 ± 0.49	2.65 ± 0.41	0.993
Sensory distance (mm)	Affected	122.50 ± 9.10	121.61 ± 10.77	123.17 ± 7.35	0.581
	Unaffected	134.60 ± 11.0	129.72 ± 8.88	138.25 ± 11.86	0.014*
Sensory conduction velocity (m/s)	Affected	45.50 ± 5.80	47.78 ± 6.33	43.82 ± 4.97	0.028*
	Unaffected	53.10 ± 8.30	54.29 ± 6.92	52.03 ± 9.49	0.397
Motor amplitude (mV)	Affected	12.85 ± 2.89	13.07 ± 2.34	12.66 ± 3.25	0.656
	Unaffected	15.27 ± 4.79	14.72 ± 5.11	15.67 ± 4.52	0.526
Motor latency (ms)	Affected	3.39 ± 0.19	3.40 ± 0.21	3.38 ± 0.17	0.828
	Unaffected	3.48 ± 0.39	3.37 ± 0.40	3.56 ± 0.38	0.139
Motor distance (mm)	Affected	216.80 ± 19.70	220.00 ± 18.65	214.58 ± 18.83	0.360
	Unaffected	239.60 ± 13.60	240.67 ± 14.29	238.71 ± 13.02	0.646
Motor conduction velocity (m/s)	Affected	50.96 ± 3.06	50.55 ± 2.60	51.28 ± 3.35	0.450
	Unaffected	52.50 ± 3.45	53.64 ± 3.92	51.63 ± 2.85	0.061

Values are presented as mean ± standard deviation. Electrophysiological parameters of the median nerve were compared between cast and plate groups using the independent samples t-test

*significant value

## Discussion

The present study provides a comprehensive comparative evaluation of median nerve involvement following distal radius fractures treated either conservatively with circular cast immobilization or surgically with volar plate fixation. By integrating ultrasonographic, electrophysiological, and clinical-functional assessments, this study offers novel insights into the structural and functional behavior of the median nerve after different treatment strategies, with particular emphasis on subclinical nerve involvement.

The principal finding of this study is that patients treated with volar plate fixation exhibited more pronounced ultrasonographic and electrophysiological alterations of the median nerve compared with those treated conservatively with casting, despite comparable clinical functional outcomes. Specifically, the plate group demonstrated a higher frequency of positive Tinel test results and night pain, increased position-dependent ultrasonographic median nerve dimensions, and a significant reduction in sensory nerve conduction velocity on the affected side. Collectively, these findings suggest the presence of subclinical median nerve involvement associated with volar plating that does not necessarily translate into overt functional impairment in the mid-term.

The position-dependent nature of these findings may reflect dynamic mechanical influences on the median nerve during wrist motion.

Ultrasonography has increasingly been recognized as a sensitive modality for detecting early morphological changes of the median nerve. Previous ultrasonographic studies have suggested that subtle morphological and mechanical alterations of the median nerve may be detected after volar locking plate fixation of distal radius fractures, even in patients without clinically apparent neuropathy [9]. In addition, ultrasonography-assisted evaluations have demonstrated an association between increased volar plate prominence and enlargement of the median nerve cross-sectional area, without concurrent deterioration in functional outcome scores at mid-term follow-up [10]. The present findings are consistent with these observations and support the concept that ultrasonographic changes may reflect early adaptive or stress-related responses of the median nerve to local anatomical and spatial alterations at the volar wrist. These findings may reflect altered local biomechanics at the volar wrist following fracture treatment.

A key strength of the current study is the direct comparison between operative and non-operative treatment modalities. Unlike previous investigations focusing exclusively on surgically treated cohorts, the inclusion of a conservatively treated cast group allowed differentiation between implant-related effects and changes attributable to fracture-related edema or immobilization alone. The more pronounced position-dependent median nerve morphological changes observed in the volar plate group may reflect local mechanical and anatomical factors; however, the potential contribution of fracture severity and treatment selection bias should also be considered.

Electrophysiological assessment further strengthened these observations. While motor nerve conduction parameters remained preserved, the reduction in sensory nerve conduction velocity observed on the affected side in the plate group is consistent with early demyelinating changes rather than established axonal injury. Similar subtle sensory conduction alterations following volar plate fixation of distal radius fractures have been reported in previous electrophysiological studies, even in the absence of clinically overt carpal tunnel syndrome [14]. The preservation of functional outcome scores despite objective findings suggests that these alterations may remain subclinical in the mid-term.

The dissociation between ultrasonographic, electrophysiological, and clinical findings observed in this study aligns with prior observations in the carpal tunnel syndrome literature. Previous studies have highlighted that ultrasonographic enlargement of the median nerve does not always correlate with electrophysiological severity or symptom burden, particularly in early or subclinical stages of nerve involvement [15]. This phenomenon may explain why objective structural and neurophysiological alterations identified in the present study were not accompanied by significant differences in patient-reported functional outcomes.

From a clinical perspective, these findings have important implications. The presence of subclinical median nerve involvement after volar plate fixation suggests that routine prophylactic carpal tunnel release may not be necessary in the absence of clear clinical indications. Instead, targeted postoperative surveillance incorporating focused clinical examination and, when appropriate, adjunctive ultrasonographic or electrophysiological assessment may facilitate early detection of nerve compromise. At the same time, the preservation of functional outcomes supports the continued use of volar plating in appropriately selected patients when performed with careful attention to implant positioning and soft tissue handling.

The strengths of this study include its comparative design, the combined use of ultrasonography and electrophysiology, and the standardized timing of assessments at 12 months post-treatment, minimizing the influence of acute post-traumatic edema and transient neurapraxia. Nevertheless, several limitations should be acknowledged.

The retrospective and single-center nature of the study may limit generalizability, and the relatively modest sample size may reduce the ability to detect smaller effect sizes, particularly in electrophysiological parameters. In addition, multiple ultrasonographic parameters were evaluated across different wrist positions and between affected and unaffected sides. Because of the exploratory design and limited sample size, formal correction for multiple comparisons was not applied, which may increase the risk of type I error. Therefore, the findings should be interpreted cautiously and considered hypothesis-generating.

Furthermore, although multivariable adjustment could potentially improve control of confounding factors, the relatively limited sample size increased the risk of overfitting and unstable estimates in additional regression-based analyses.

Formal fracture classification according to the AO/OTA system was not systematically available because of the retrospective nature of the study, limiting the ability to fully control for fracture severity between treatment groups. Additionally, longer-term follow-up is required to determine whether the observed subclinical changes progress to clinically relevant neuropathy over time. The exclusion of Soong type 3 plates may limit the generalizability of the findings to cases with extreme volar plate prominence; however, this approach allowed for a focused assessment of median nerve behavior in patients with standard plate positioning. Additionally, the absence of fracture classification data and the non-randomized treatment allocation introduce potential confounding, which should be considered when interpreting the results.

Overall, the present findings contribute to a better understanding of median nerve behavior following distal radius fracture treatment. The observed ultrasonographic and electrophysiological alterations may reflect local anatomical and mechanical influences on median nerve behavior. From a clinical standpoint, these findings suggest that objective neurophysiological assessment may complement symptom-based evaluation in detecting early median nerve involvement after volar plating.

## Conclusion

This comparative study showed that patients treated with volar plate fixation exhibited more pronounced subclinical ultrasonographic median nerve alterations and subtle electrophysiological differences compared with those treated conservatively with cast immobilization. However, because of the retrospective and non-randomized design, these findings should be interpreted as associative rather than causal.

Despite these objective findings, clinical examination results and patient-reported functional outcome scores were comparable between treatment modalities at the 12-month follow-up. These results suggest that median nerve alterations following volar plate fixation may remain subclinical in the mid-term and do not necessarily translate into measurable functional impairment.

The findings of this study suggest that combined ultrasonographic and electrophysiological assessment may be useful for detecting early median nerve changes after distal radius fracture treatment. Rather than routine prophylactic intervention, careful clinical follow-up supplemented by targeted neurophysiological evaluation may be more appropriate in patients treated with volar plating. Further prospective studies with larger cohorts and longer follow-up are warranted to clarify the long-term clinical relevance of these subclinical findings.

## Data Availability

No datasets were generated or analysed during the current study.
